# A Bioecosystem Theory of Negative Symptoms in Schizophrenia

**DOI:** 10.3389/fpsyt.2021.655471

**Published:** 2021-03-25

**Authors:** Gregory P. Strauss

**Affiliations:** Department of Psychology, University of Georgia, Athens, GA, United States

**Keywords:** environment, ecological systems, psychosis, network, development

## Abstract

**Objective:** Negative symptoms are a core feature of schizophrenia that has been linked to numerous poor clinical outcomes. Although person-level mechanisms have been identified for negative symptoms, psychosocial and pharmacological treatments targeting these mechanisms have been ineffective. The current theoretical paper proposes that limited treatment progress may result in part from a failure to identify and target environmental processes that cause and maintain negative symptoms.

**Methods:** A novel theoretical model is outlined, called the bioecosystem theory of negative symptoms, that offers a conceptual framework for studying interactions among environmental systems and person-related biological and psychosocial factors.

**Results:** Relying on Bronfenbrenner's developmental theory as an organizing framework, four interactive environmental systems are proposed to be critical for the genesis and maintenance of negative symptoms: (1) Microsystem: the immediate environment; (2) Mesosystem: the interactions among microsystems; (3) Exosystem: indirect environments that influence the individual through the microsystems; (4) Macrosystem: socio-cultural factors. The environmental factors within these systems are proposed to function as a network and have dynamic within-system interactions, as well as cross-system interactions that change over time and across phases of illness.

**Conclusions:** Environmental contributions to negative symptoms have received minimal empirical attention, despite their potential to explain variance in negative symptom severity. The bioecosystem model of negative symptoms introduced here offers a novel conceptual framework for exploring environmental contributions to negative symptoms and their interaction with person-level biological and psychological factors. This theory may facilitate new avenues for identifying environmental treatment targets and novel systems-level interventions.

## Overview

Schizophrenia (SZ) is associated with high rates of functional disability worldwide (1). Negative symptoms (alogia, blunted affect, anhedonia, avolition, asociality) are the strongest predictor of poor functioning and associated with numerous other detrimental clinical outcomes (e.g., illness liability, low rates of recovery, reduced subjective well-being) (2). Although negative symptoms are clearly a critical treatment target, available pharmacological and psychosocial interventions have not lead to clinically meaningful improvement (3).

To date, the field's approach to identifying negative symptom treatment targets has involved examining psychosocial and biological factors within the person, such as beliefs/attitudes, cognitive/reward processes, and neural mechanisms (4). Although such investigations have led to invaluable knowledge regarding processes that contribute to negative symptoms within-individuals, this approach has not led to significant treatment breakthroughs. Why is this? Have we not identified the right targets? Have we identified key targets but the interventions attempted have not adequately augmented their underlying mechanisms? Or have the critical person-level targets been identified and appropriate treatments developed, but factors external to the person are preventing symptomatic improvement?

One underexplored explanation for the limited progress in treating negative symptoms is that there are strong environmental factors at play that have yet to be targeted. If such environmental factors do exist and account for a sizable proportion of variance in negative symptoms, this may explain in part why pharmacological and psychosocial treatments have proven ineffective, i.e., environmental factors would need to be augmented prior to or in conjunction with person-focused treatments.

In this theoretical paper, I propose that a new approach to studying negative symptoms may lead to valuable insights into environmental factors that cause and maintain negative symptoms. Relying on the ecological theory proposed by Bronfenbrenner (5–8) as an organizing framework, a conceptual model is outlined for exploring person and environmental systems level interactions in the genesis and maintenance of negative symptoms: the bioecosystem theory of negative symptoms. Just like Bronfenbrenner's original theoretical papers (5, 6), this manuscript is not meant to be an exhaustive review of a literature that is already established and used to support a theory. Rather, the aim is to put forth a conceptual framework that can guide scientific exploration needed to evaluate the theory via specific testable hypotheses. Recommendations regarding methods for studying ecological systems are also proposed and potential future treatment implications are discussed.

## Bronfenbrenner's Model

Throughout his career, Bronfenbrenner (5–7) revised his “Ecological Model of Human Development” several times. Each iteration was designed to explain how individuals change within their environments throughout the lifespan. The final model introduced the Process-Person-Context-Time (PPCT) framework (8), which has continued to influence the field until the present. Broadly, this model espoused that a person exists within an inter-connected network of relationships, activities, roles, and settings. Individuals are active agents within these environmental systems, through which they learn new mental structures and roles that promote development. At the same time, the systems around the individual change according to the person's actions in a cycle of bi-directional influences that shape development.

### Process

A key aspect of this model is the concept of proximal processes, which Bronfenbrenner considered the “engine of development.” Proximal processes are enduring forms of interaction between the individual and their immediate environment (i.e., the microsystem) and the means by which genotypes transform into phenotypes (i.e., how specific sets of genes turn into observable psychological, behavioral, and physical characteristics via their interaction with the environment). Engagement in activity within the microsystem is critical in this transformation, fueling development and change. Activities that the individual engages in become increasingly more complex across time as the person interacts with people and objects in their settings. Thus, being an active (rather than passive) agent in one's microsystems is key to the development of normative social, emotional, behavioral, biological and other processes.

### Person

The person component of the model focuses on genetic, neural, and other biological factors that have dynamic interactions with the microsystem. Bronfenbrenner defined three key characteristics related to the person that influence their interactions within microsystems: demand, resource, and force. Demand characteristics influence immediate interactions/reactions that others have to the person within an environment (e.g., age, skin color, sex, physical appearance). Resource characteristics involve cognitive, social, emotional, and material resources a person possesses that influence their interactions within the microsystem. Force characteristics involve individual differences in temperament, motivation, and persistence. Individual differences in these characteristics have an important influence on development and how an individual adapts to their ever-changing environment. For example, an individual may have adequate resource characteristics, but if force characteristics are diminished they may not have the motivation to engage or persist in activities within the environment that are key to acquiring new skills and abilities that drive change and development.

### Context

**Figure 1:**
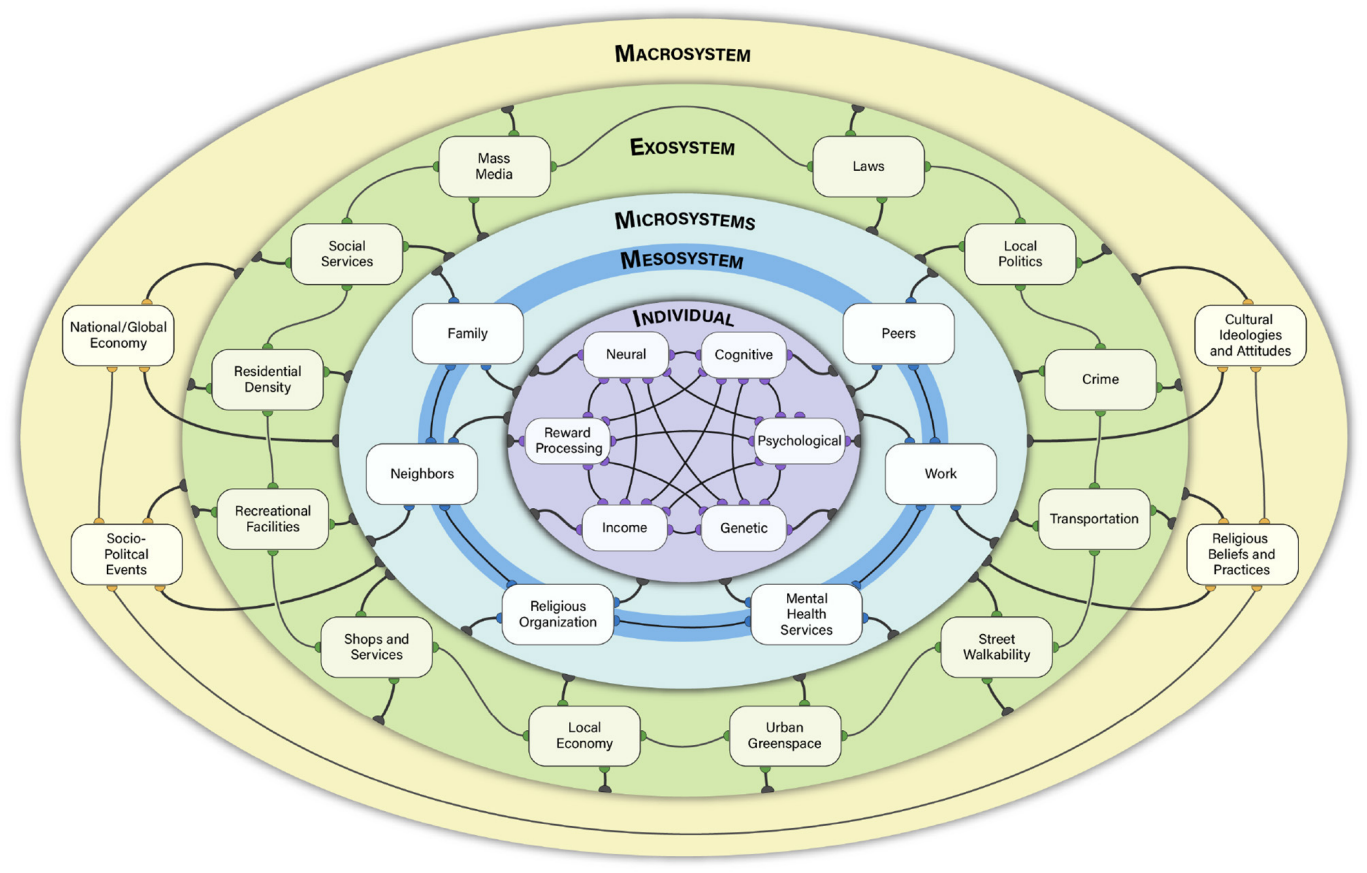
Bioecosystem model of negative symptoms conceptual overview. In the bioecosystem theory, negative symptoms are influenced by dynamic interactions between person-level factors and environmental systems. The environmental systems differ in their proximity to the individual. The Microsystem represents the immediate environment. Multiple microsystems can exist (e.g., home, work, school). The mesosystem refers to connections or interactions between various microsystems (e.g., a friend from work also goes to the same church as the individual). The exosystem represents the indirect environment, which has the potential to act on the person. The Macrosystem reflects sociocultural factors. These systems are proposed to function as a dynamic and interactive network, with different levels of the ecosystem having differing influences on negative symptoms throughout phases of illness and development. Yellow, Macrosystem; Green, Exosystem; Light Blue, Microsystem; Dark Blue, Mesosystem; Purple, Person-Level Factors. Connections among the elements within the ecosystem are meant to be illustrative of potential network level interactions, rather than an exhaustive list of possibilities.

**Figure 2:**
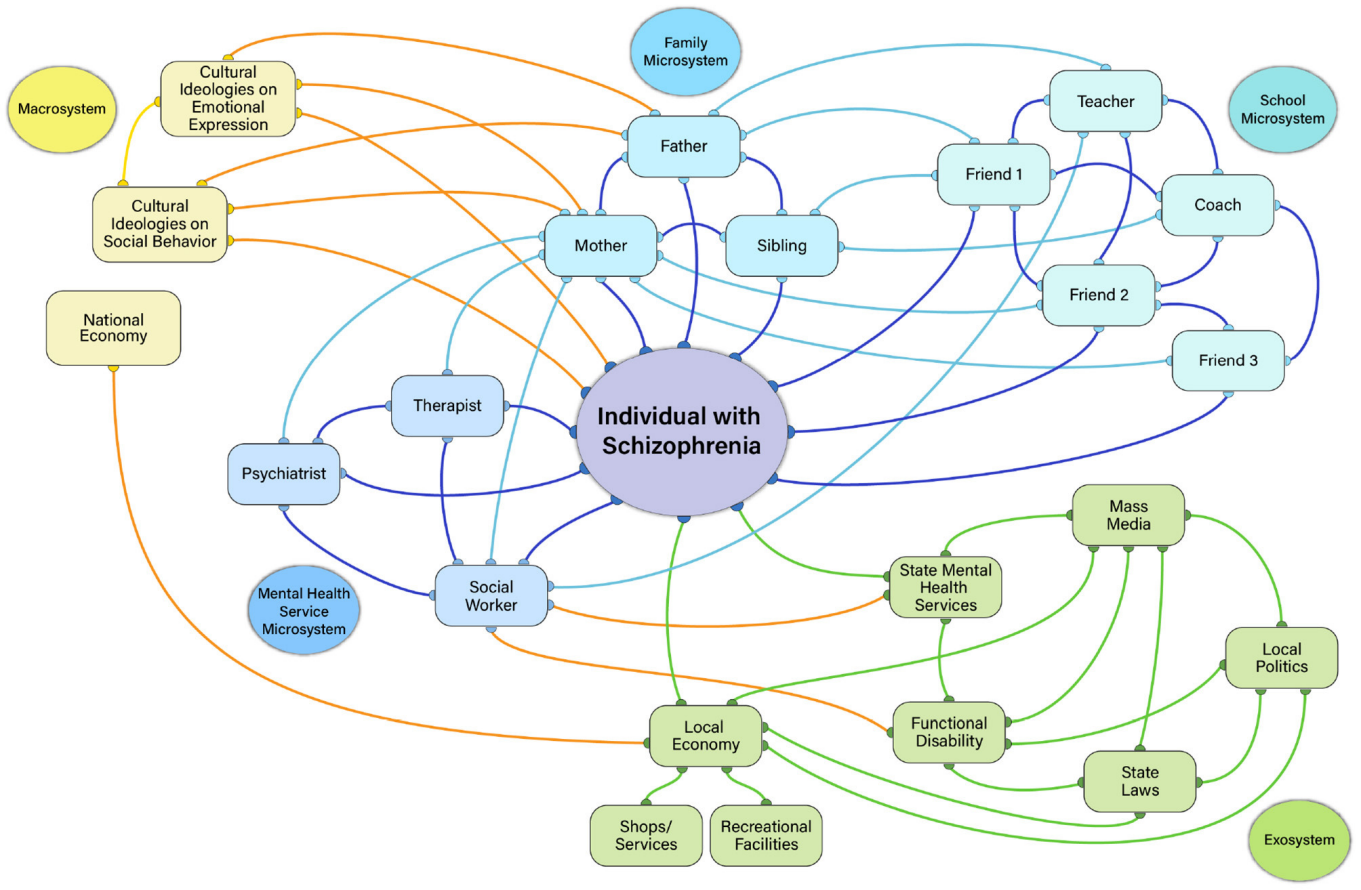
Low negative symptom ecosystem network illustration. In this example, imagine an 18 years old male who is in the first episode of schizophrenia with low negative symptoms. He has three prominent microsystem settings: home, school, mental health services. In the home setting, he has strong social ties with his mother, father, and brother, whom are all highly connected to each other. The mother is a highly central driving force in the individual's microsystem, connecting them (mesosystem) with their mental health services (taking them to appointments with psychiatrist, therapist, social worker, and being part of the treatment) and school (interacting with teachers, coaches, and their friends). The density of connections within and across microsystems, as well as the mother's high centrality within the network is protective against developing negative symptoms. The family is also deeply connected with their culture (macrosystem): the mother, father, and individual with the illness have all adopted beliefs of their ethnic culture regarding emotional expression, socialization, work/motivation, and pleasurable activities. Strong cultural identification and connection with others of the same culture in the neighborhood and school are protective against negative symptoms. At the level of the exosystem, the individual lives in an affluent suburban area that is rich in resources for social, pleasurable, and goal-directed activities (e.g., recreational centers, shops) that are accessible (they have a car, can walk to facilities, there is low crime). Exosystem factors are not prominently acting on the person. They do not receive functional disability benefits from the state, although these are available. Local politics and state laws are less influential on this person because they are not receiving disability services. Yellow, Macrosystem; Green, Exosystem; Light Blue, Microsystem; Dark Blue, Mesosystem; Orange, cross-system interactions; Purple, Person-Level Factors.

**Figure 3:**
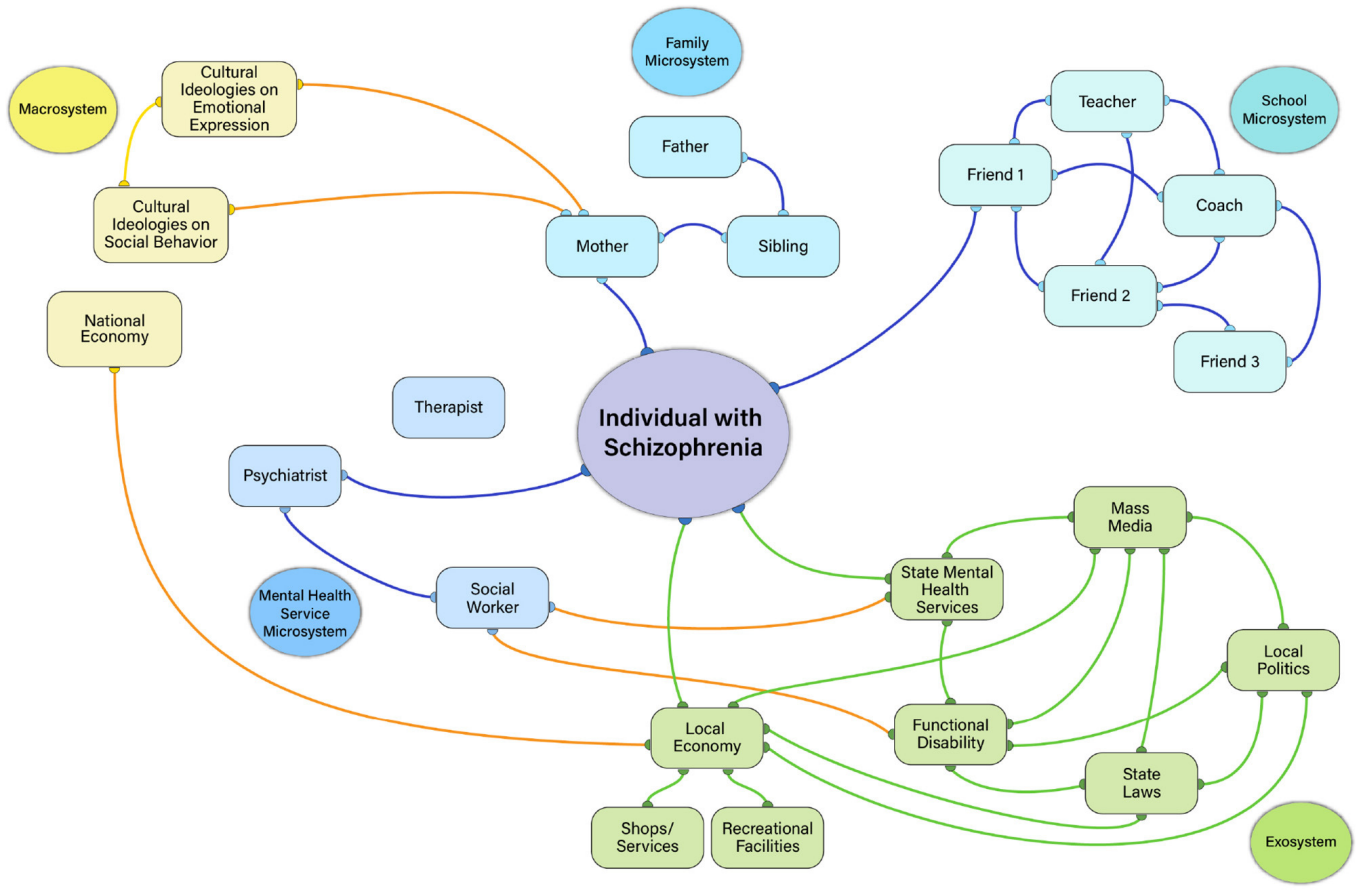
High negative symptom ecosystem network illustration. In this example, imagine an 18 years old male who is in the first episode of schizophrenia with high negative symptoms. Just like the example in Figure 2, he has three prominent microsystem settings: home, school, mental health services. However, unlike the low negative symptom example in Figure 2, he is not densely connected to the people and places within these settings (note fewer orange lines within microsystems). He interacts with his mother, but does not interact with his father and brother, even though his mother, father, and brother all interact with each other. Unlike Figure 2, there is no highly central figure helping to make connections across microsystems (i.e., the mesosystem has low density- note the absence of yellow lines). As a result, he is less likely to engage in as many close social interactions or recreational activities with his peers or family. He has a psychiatrist, but is not connected with a therapist or social worker to coordinate care. The mental health professionals in the local mental health setting are also generally less connected to each other and functioning independently. As such, he is not receiving the same quality of care as the patient in Figure 2 and is less motivated to receive treatment. He has stopped going to school. He maintains contact with one friend from school, who has connections to other friends of their own, teachers, and coaches. But, the individual themselves no longer maintains ties with those in the school setting beyond the one friend. At the exosystem level, the patient lives in poverty stricken area with few resources (shops, recreational facilities), high levels of crime, and poor transportation access. These factors contribute to less socialization, recreational activities, and goal-directed activities. He receives disability benefits and has just enough money to live in a subsidized group home, but not enough to socialize frequently or pursue recreational activities. Local politics and state laws/policies influence details related to the disability benefits, subsidized housing, and mental health services the patient receives. Yellow, Macrosystem; Green, Exosystem; Light Blue, Microsystem; Dark Blue, Mesosystem; Orange, cross-system interactions; Purple, Person-Level Factors.

Bronfenbrenner proposed the existence of a set of four hierarchically organized ecosystems that vary in terms of how proximal they are to the individual (see Figure 1 for an adaptation) (5–7). These systems include the: (1) Microsystem: immediate environments and contexts that have a direct influence on an individual's activities, roles, and social interactions (e.g., home, school, workplace, religious community). (2) Mesosystem: the interactions among microsystems; (3) Exosystem: indirect environments that influence the individual through the microsystems (e.g., economics, government, laws, political systems, mass media); (4) Macrosystem: social ideologies, norms, and values of the culture and sub-cultures (e.g., cultural display rules for emotional expression, norms for social interactions). The environmental factors within these systems were proposed to have within- and cross-system interactions. The dynamic interactions among systems are critical, with bi-directional influences of the microsystem thought to have the strongest impact on the individual and the more distant structures influencing the person indirectly via their impact on the microsystem. Although Bronfenbrenner originally described these systems as being hierarchically organized similar to a set of nested Russian dolls, more modern perspectives have conceptualized them as networks that have within and between systems interactions (9) (see Figures 1–**3**).

### Time

Bronfenbrenner introduced the “chronosystem” to capture changes across time that can occur not only within the person (e.g., neural maturation), but also the broader ecosystem, e.g., microsystem (e.g., individuals in one's immediate home and school environment change), mesosystem (e.g., the advent of the internet and social media which allowed more rapid communication within an across miscrosystems), exosystem (e.g., changes in state laws occurring with transitions in political office), and macrosystem (e.g., a culture integrating more accepting views of another culture over years) (7).

Bronfenbrenner's PPCT model has received substantial support in the field of developmental psychology and become one of the most influential conceptual frameworks (e.g., as of 10/06/2020, the original conceptual treatise has been cited >48,000 times on google scholar) (5). Decades of research also demonstrate how Bronfenbrenner's framework has been invaluable for understanding physical (e.g., diabetes) and mental illnesses (e.g., conduct disorder, suicide, social anxiety, trauma) that exist as interactive bioecosystems (10–13). More recent frameworks have expanded on Bronfenbrenner's concepts, demonstrating that systems approaches applied to psychology and public health have identified environmental drivers of health outcomes that are multi-faceted, diverse, and inter-related (14). For example, numerous mental and physical health problems are associated with aspects of the “built environment” (i.e., human-modified/created places such as homes, workplaces, industrial areas, parks, schools, roads), which can be augmented by creating sustainable communities (15). Given the complex interplay between multiple environmental systems on the built environment (e.g., social, political, economic), public policies aimed at improving health outcomes have necessitated a very different approach than the person-focused efforts common to psychology/psychiatry; they have required multi-level community-based approaches that involve a multi-disciplinary team capable of enacting change across the varied systems leading the environment to influence health (e.g., politics, social reform, connecting healthcare providers with policy makers).

To date, the fields of psychiatry and psychology have yet to systematically explore environmental influences on negative symptoms or develop ecosystem focused interventions for them. However, given the importance of environmental factors in other psychiatric disorders and general effectiveness of systems-level interventions, it may prove useful to adopt such a focus for negative symptoms.

## A Bioecosystem Model of Negative Symptoms in Schizophrenia

Although there has been considerable progress in identifying environmental processes associated with onset and maintenance of psychotic disorders (e.g., immigration, urbanicity, ethnic density, early life adversity, expressed emotion in the home) (16), few studies have examined environmental contributions to negative symptoms specifically. However, there is some suggestion that certain environmental deprivation factors are associated with negative symptoms, including under-stimulating environments (17, 18), smaller social networks (19), aberrant family social dynamics (e.g., cohesion, positive emotion expression) (20, 21), greater local income inequality (22), lower socio-economic status (23), urbanicity (24), and receiving minimal care and attention in group homes (25).

Although the aforementioned evidence provides modest suggestion that environmental factors relate to negative symptoms, no unifying conceptual framework has been proposed to systematically explore these factors. Below I propose such a framework based on Bronfenbrenner's PPCT model, called the bioecosystem theory of negative symptoms. This framework is overviewed in Figure 1. As depicted in the Figure, the individual is at the center, with known person-level factors influencing negative symptoms at the heart of the model. Influencing the person are environmental systems of varying degrees of proximity, including the microsystem, mesosystem, exosystem, and macrosystem. Although these environmental systems are the novel part of the model, I do not view them as more important than the person-level factors. Rather, biological, psychological, and environmental systems-level factors function within a dynamically interactive network; these factors have differing degrees of connection and influence across individuals, time, and phases of illness.

## Person-Level Factors

Several person-level factors have been identified as core negative symptom mechanisms (4). For example, avolition and anhedonia been associated with dysfunctional cortico-striatal interactions that impact numerous aspects of reward and emotion processing needed to drive motivated behavior (e.g., anticipatory pleasure, reinforcement learning, effort-cost computation) (26, 27). Blunted affect and alogia have been associated with cognitive impairments, such that overly taxed cognitive resources induce reductions in speech and facial emotion (28). Primary and enduring negative symptoms have been associated with white and gray matter abnormalities (29). Negative symptoms have been tied to genes (e.g., NKAIN2, LSM6, BCAT1) and ontological pathways associated with neuron projection (30), as well as other biological correlates (e.g., inflammatory cytokines, glucose tolerance, oxidative stress) (31–33). Psychological factors, such as defeatist beliefs, asocial beliefs, and low pleasure beliefs have been associated with avolition, anhedonia, and asociality (34–36). Socio-demographic factors have also been associated with negative symptoms (e.g., male sex, lower income, summer birth) (37). Several of these factors have been associated with negative symptoms not only during the chronic phase of illness, but also in the prodromal phase, suggesting that they may be involved with the emergence of negative symptoms and risk for conversion to overt illness (38–42). Using Bronfenbrenner's terminology, these biological and psychological person-level factors would determine an individual's demand, resource, and force characteristics and may influence the quality or quantity of their microsystems.

## Environmental Systems That may Contribute to the Onset of Negative Symptoms

There are likely multiple developmental pathways to negative symptoms that are influenced by interactions between person-level and environmental factors. To understand how negative symptoms emerge and are maintained, it will be necessary to understand development within context. Has the environment been static across development or have there been critical changes within the ecosystem at pivotal points in childhood, adolescence, or adulthood? Answering this question may be critical for determining in which phase of illness negative symptoms emerge, as there is considerable variability across individuals and evidence that they can begin in toddlerhood, childhood, adolescence, early adulthood, middle adulthood, and late adulthood.

Although the premorbid phase is typically considered a period devoid of psychopathology, this is a misnomer with regard to negative symptoms. Seminal work by Elaine Walker et al. (43) that used a retrospective home video sibling-pair study design found that young children who later go on to develop schizophrenia have diminished facial affect and social behavior. What might contribute to the emergence of these symptoms during the premorbid phase? Certainly person-level biological (genetic, neural, cognitive) or temperamental factors might play a role. However, it is also possible that environmental factors in the microsystem could interact with these processes, such as impoverished immediate environments with few resources (material, person) needed to promote activity. Bronfenbrenner (8) considered engaging in activity particularly important to development during early years. He proposed that activity must be frequent and long enough and lead to activities that are increasingly more complex. Participation in such interactive processes over time generates the ability, motivation, knowledge, and skill to engage in social, recreational, and goal-directed activities with increasing independence. Throughout development, individuals typically increase their level and range of capabilities; to continue progressing, it is necessary for proximal processes to also continue evolving. Without this synergistic increase in complexity between activities the individual attempts and resources within the environment expanding to match their advancement, development can slow or reverse direction. Whether impoverished immediate environments are common in formative early years of development among those who later go on to develop schizophrenia has yet to receive empirical attention.

Another pathway to negative symptoms could emerge during the prodromal or first episode periods. These individuals may develop skills and behavior normally until a certain point and then regress after environmental complexity reduces. For example, imagine a scenario where an individual experiences an emergence of attenuated or fully psychotic symptoms at age 18. Development could have progressed typically up until that point; however, after symptoms emerge, interactions with other members of the ecosystem change because their view of the person changes. They gradually come to ascribe a new role to the person experiencing psychosis. With the change in role to person with a serious psychiatric illness comes a shift in interaction patterns within the environmental systems. Relatives, friends, and significant others may alter or reduce their level of interaction. Or the person themselves may withdraw from their networks at an inopportune time in development when youth are taking on new roles, exploring new settings, and expanding their microsystems. The person with emerging or newly onset psychosis may be left behind, leading to a less connected microsystem and fewer opportunities for engaging in social and goal-directed activities. A halting of or regression in the complexity of roles and activities may further exacerbate negative symptoms, leading the person to become even less motivated to explore, maintain, or transform the ecosystem. They may then become less likely to adapt to and function in the ecosystem, maintaining biological and psychological (e.g., defeatist beliefs) processes that underlie negative symptoms and forming a self-sustaining feedback loop that promotes chronicity. Consistent with this notion, preliminary evidence suggests that those at clinical high-risk for psychosis report less social support and that this is associated with greater symptomatology (44).

Other examples may include instances where negative symptoms do not develop until the illness has been established for several years in adulthood. For example, where an individual did not display negative symptoms until after their environment becomes impoverished in terms of material, financial, microsystem (e.g., density of social networks), and exosystem (e.g., access to recreational centers, transportation, healthcare) resources that occurs with increasing chronicity and age. Evidence for an association between greater negative symptom severity during the chronic phase of illness has been associated with reduced social network size, social cohesion, quality of health care, financial resources, and elements of the indirect environment (17–25). However, since the evidence is cross-sectional, it is unclear whether these factors lead to the development of negative symptoms or if they are a byproduct of them.

## Environmental Systems That may Contribute to the Maintenance of Negative Symptoms

### Microsystem and Mesosystem

How might the microsystems (i.e., immediate environments) and mesosystem (i.e., connections among microsystems) contribute to the maintenance of negative symptoms once they have already emerged? A key tenet of Brofenbrenner's model is that individuals are active agents within their microsystems. The activities they engage in are critical for facilitating growth and developing new roles that allow them to expand into new settings. However, this assumption may not be valid for those with negative symptoms who are by definition more passive participants within the environment. An expected outcome of such passivity would be a reduction in the total number of distinct microsystems and the inter-connections among microsystems (i.e., the mesosystem).

To operationalize these microsystem and mesosystem abnormalities it is helpful to view them from a network perspective. The field of network science has identified a number of relevant constructs and mathematical formulations that are used to understand dynamic system interactions (46). One key network concept is density, i.e., the level of global inter-connectedness within a network (47). Those with negative symptoms would be expected to have less densely connected microsystems and lower density of connections across microsystems. Although prior studies have indicated that more dense networks are associated with greater increases in psychopathology much of the time (47), negative symptom microsystems may reflect a unique instance where less densely connected networks are more pathological as they reflect aberrant reductions in thought/emotion/behavior rather than pathological excesses.

To illustrate such possibilities, consider the hypothetical networks in Figures 2, 3, which depict a person with low (Figure 2) compared to high (Figure 3) negative symptoms. As shown in Figure 2, the individual with low negative symptoms is highly connected to each of the people within each microsystem, who are highly connected with each other. In contrast, the person with high negative symptoms in Figure 3 has access to the same types of people in their individual microsystems, but is not interacting with them as much within each setting. The individuals within their various microsystems may have connections with each other, but the global number of connections across microsystem settings is reduced. The density of connections among microsystems may be critical for influencing whether an individual with schizophrenia has the tools to explore other aspects of the environment.

Another relevant network concept is centrality, which refers to how influential a person is within their microsystem. One might expect those with high negative symptoms to be less central within their own microsystem networks than someone without negative symptoms (i.e., less likely to be the key point of interaction driving communications and actions). However, it is also possible to use centrality metrics to identify whether other individuals are highly influential in an environment and who those persons are. There are multiple ways in which centrality of others in the environment could contribute to negative symptoms. Consider the network depicted in Figure 3. The high negative symptom patient has no central figure linking them within and across settings. This may be a common example for the microsystems of outpatients in the chronic phase of illness who are likely to live apart from their families in group homes and have limited interactions with individuals in other settings. Essentially, their negative symptoms are influenced by having no/few individuals within their microsystems who have high centrality. However, another common scenario involves cases where the patient has an individual who is highly central within their environment. For example, consider the case of a mother who is highly motivated to help her son who has recently developed the illness reestablish social ties, attend school, and purse recreational activities. She provides the impetus for her son's activities, pushing him to complete activities such as homework, receiving mental health treatment, and attending church. Although well-intentioned, the mother's high centrality in this network may inadvertently contribute to experiential negative symptoms, removing the patient's autonomy and decision-making, further reducing his motivation to independently pursue activities, socialize, and engage with the environment. Very high or very low centrality may therefore relate to negative symptoms.

Another critical feature of microsystems is how rich they are in physical and material resources. Bronfenbrenner described their importance, noting that:

“*Proximal processes are not limited to interactions with people; they also can involve interaction with objects and symbols. In the latter circumstance, for reciprocal interaction to occur, the objects and symbols in the immediate environment must be of a kind that invites attention, exploration, manipulation, elaboration, and imagination.”* (p. 798) (40).

Many individuals with schizophrenia have lower incomes and live in impoverished or under-stimulating environments. Physical characteristics of the settings people with schizophrenia live in (i.e., environmental richness) may be less conducive to constructing complex activities. Impoverished microsystem settings may reduce opportunities for reciprocal interactions with the environment, thereby diminishing exploration and the ability to generate mental representations of future activity needed to initiate behavior. Under-stimulating or impoverished immediate personal environments may also be intimately related to indirect environmental systems within the exosystem or macrosystem (e.g., low personal income is driven by an impoverished local or global economy; impoverished resources for performing recreational activities in the home may be related to the local/global economy and a “built environment” that lacks resources for enriching the immediate environment).

### Exosystem

In addition to microsystems and their connecting mesosystem, there are likely to be other environmental systems that make important contributions to negative symptoms. One of those is the exosystem, which denotes the indirect environment that the individual does not actively participate in, but is still affected by. As depicted in Figure 1, there are many plausible exosystem factors that could influence negative symptoms. Exosystem factors may have at least two possible connections to the person with negative symptoms: by influencing what happens to the person in the ecosystem or what is available to them in terms of resources for activities.

Some elements of an exosystem may limit resource availability. Limited availability of resources, rather than limited desire alone, may influence how often some individuals perform recreational, role, and social activities. For example, consider exosystem elements in Figure 1. Factors such as transportation availability and street walkability could influence whether a person is able to engage in recreational pursuits if they do not have access to a car. Imagine that they live in a rural environment with no sidewalks and there is no public bus/train system that is accessible. Such factors would limit which activities an individual could engage in and how easily they could socialize with others. Proximity to quality resources may also be critical. For example, an individual may live in an impoverished area with few recreational centers, shops, restaurants, or greenspace. Residential density may also influence how many individuals the person has access to interacting with or how feasible it is to be gainfully employed (e.g., too much competition for local jobs during a poor economy). The amount and type of crime in one's neighborhood may influence how often individuals socialize or engage in activities outside of their home.

Other aspects of the exosystem may dictate what transpires in microsystem settings. These “power settings,” as (48) referred to them, would indirectly impact people with schizophrenia by influencing politics and laws related to mental health services, disability policies, workplace discrimination against those with mental illness, subsidized housing, and vocational rehabilitation. How these power settings are linked to the mesosystem may impact whether microsystems contribute to negative symptoms. For example, consider the illustrations in Figures 2, 3. In both Figures, state laws and local politics are intricately linked to functional disability and the hypothetical patient's mental health service microsystem. However, only the high negative symptom patient is receiving functional disability in these examples, and the state laws/local politics have an indirect effect on the patient through their connection to disability. Imagine that this person is living in one of the US states with laws that are less protective of those with mental health diagnoses from being discriminated against in the workplace. This individual may have a harder time being hired, get fired, or receive unfair treatment on the job due to their illness- all factors that could promote limited financial resources, reduced activity, and reduced socialization. Or consider that supportive employment services are not paid by all U.S. state Medicaid programs, limiting opportunities for engaging in an important aspect of goal-directed activity (i.e., gainful employment). The politics driving decision-makers within “power settings” may have an indirect effect on the person with schizophrenia by influencing their access to jobs, income level, affordable housing, whether they are eligible for disability benefits, and which mental health services can be accessed. Such factors, in turn, may contribute to negative symptoms.

### Macrosystem

The macrosystem, which entails cultural beliefs, ideologies, and attitudes, would be expected to have a cascading effect on the other layers of the ecosystem. For example, cultural beliefs may affect the structures in which the patient functions, how those in the immediate environment engage the patient, which social interactions are prominent and valued, how emotions are experienced and expressed, and which goals an individual pursues. There is widespread evidence for cultural variations in attitudes/beliefs that influence norms regarding social activity, emotion, and behavior. For example, certain Asian cultures are known to highly value low arousal positive emotion (e.g., contentment), whereas Western cultures value high arousal positive emotions (e.g., excitement) (49). Norms regarding emotional expression also differ across these cultures, influencing how intensely, frequently, and in which contexts it is normative to display emotion (50). If an individual with schizophrenia has not learned these social norms or does not value them (e.g., due to social cognition deficits), would this individual be likely to display affect at the appropriate times or cultivate the activities that would bring them pleasure in a way that is consistent with their culture's expectations? It is possible that failing to adhere to these cultural ideologies, attitudes, and norms may contribute to the manifestation of negative symptoms in specific contexts.

It is also possible that the individual's culture interacts with the culture of the immediate environment to promote negative symptoms. One construct relevant to this notion is “ethnic density” (i.e., whether the individuals in the immediate environment are of the same or different ethnicity to the individual) (51). If people in the immediate environments of those with schizophrenia (e.g., home, work, school, neighborhood, mental health center) are of the same culture (e.g., race, generation), this may promote within-culture views on emotion, social, and motivated behavior. Cultural congruence with the environment may facilitate acceptance and less stress around adopting views of the dominant culture. As a minority, living in a neighborhood which has a high density of people from the dominant culture may restrict the degree of connectedness of the microsystem. In turn, this may exacerbate negative symptoms by reducing the amount of connections across microsystems.

Additionally, interactions with those of a different race/ethnicity may contextually produce negative symptoms that are less likely to be present when individuals are not interacting with those of their own race/ethnicity. For example, imagine a scenario where a Black person with schizophrenia attends a day program in a predominantly White area of town, with all White clinicians, and all White consumers participating in their treatment services. The cultural incongruence of the context may lead them to speak less (alogia), be less motivated to form close social relationships (asociality), and less interested in attending the day program (avolition). A culturally consistent or congruent macrosystem makes roles, relationships, and activities easier to navigate because they are more predictable (52). Cultural incongruence may contribute to negative symptoms. Minimal studies have explored the effect of cultural congruence on negative symptoms; however, there is some evidence that White clinicians judge negative symptoms to be higher in Black individuals with schizophrenia than white individuals with schizophrenia (45). Whether this reflects cultural rater biases or contextual effects is unclear.

## Treatment Implications

Identifying environmental factors associated with specific forms of psychopathology has led to key treatment advances by spurring environmentally focused interventions. For example, multi-systemic therapy was developed for youth with conduct problems (53). In this therapy, clinicians conduct a comprehensive assessment of multiple environmental systems contributing to psychopathology. A combination of person-, family-, and systems-focused techniques are then used to address factors contributing to symptoms. The intervention is individualized to each person's unique needs, and often includes working with the family, neighbors, peers, school staff, and community organizations. Such treatments have proven efficacious in improving clinical outcomes in multiple disorders conceptualized as having both biological and environmental contributions (54). The theory also led to key educational and social reforms, such as the US Head-Start program for low income children and families.

If future studies support the notion that person- and ecosystem-level factors underlie negative symptoms and have dynamic interactions within complex networks, there may be important new treatment targets that are identified. Changes in people change microsystems, and changes in microsystems change people. As such, it is likely that interventions designed for negative symptoms will need to be multi-level, targeting elements at the person-level (e.g., via CBT, social skills, pharmacological) and systems-level (e.g., via approaches similar to MSST). Just as biological and ecosystem factors underlying negative symptoms would benefit from being viewed in a network framework, so too may treatment. The key questions for interventions may be how much effort to direct at both the individual, the setting, and their interactions? Which settings and systems are most important? This undoubtedly varies from person to person, making the prospect of a structured and standardized intervention that applies to all with the illness difficult to imagine. The functional analysis approach proposed by Lincoln et al. (55) may be useful for multi-systemic treatment case conceptualization, which would need to be personalized for each patient. At present, no validated assessment tools exist for this purpose that could be used in a clinical setting. The development of such tools that can be used to inform case conceptualization and treatment planning will be critical before systems level interventions can be implemented. Those developed for the MSST serve as a useful example.

What would one target in systems-level interventions? Which treatment components would these interventions include? Who would be involved with performing or facilitating the intervention? The answers to these questions would of course vary from person to person depending on which environmental systems are contributing to their negative symptoms and how they interact with person-level factors.

Systems-level interventions might be performed by professionals from a range of disciplines (e.g., psychiatry, psychology, social work). As validated in MSST, they would involve clinicians working within the home, work, school, and community settings and partnering with diverse community professionals such as school psychologists and religious leaders. Rather than removing the patient from their home environment (e.g., day program, inpatient hospitalization), systems focused interventions occur in the patient's daily environment. Approaches such as Assertive Community treatment, which is implemented throughout many communities in the USA, provides a useful model for how teams of professionals can work together to provide services across multiple settings.

Interventions targeting the microsystem might begin by evaluating social network density (i.e., are their too few connections within and across immediate environments) and centrality (i.e., do they lack sufficient support from key individuals or receive too much support, such that autonomy is impacted). Density might be targeted by intervention techniques such as guided peer support, the volunteer partner scheme, and supported engagement in social activity that have been shown to increase social network size (56). Centrality might be impacted by focusing on enhancing existing family relations, building friendships with peers inside and outside of current clinical services and immediate environments, and the integration of social contacts across microsystems (e.g., promoting cross-environment social interactions between those in a religious microsystem and a school microsystem). Macrosystem-level interventions might focus on helping the individual to increase access to others from the same culture if negative symptoms are driven by contextual cultural factors (e.g., cultural incongruence within the direct or indirect environment).

Exosystem-level interventions might be the most challenging to enact because they would require changes to public policies, laws, and access to mental health services. For example, if exosystem-level power settings are linked to negative symptoms, this would give rise to programs focused on changing larger political and economic policies, societal attitudes, and new programs that increase access to resources and enrich environments among those with mental illness (e.g., focused on access to transportation, safe and enriched housing, recreational facilities, shops, greenspace). It is unlikely that those with negative symptoms would be apt to influence power settings sufficiently on their own. However, mental health advocates could have a profound effect. Advocacy efforts might focus on increasing awareness among key policy-makers regarding what negative symptoms are, how indirect environments influence them, how the actions of those in power settings contribute to negative symptoms, and which policies/laws could be changed to benefit the lives of those with the illness. Advocacy for those with negative symptoms may be particularly important given that there are many intervening steps/people between them and key policy/decision-makers.

These systems focused approaches would also benefit from being complemented with person-centered approaches targeting cognition, motivation, and behavior (e.g., CBT, social skills training). It is likely that treatment will need to utilize a tiered process that prioritizes environmental systems and person-related factors at different times. For example, imagine an individual's key environmental system contributing to asociality is at the microsystem level and involves a social network that is restricted in size. Once the network has been broadened and the individual is engaging in more social contacts, they may have setbacks as they become more active in the environment and develop asocial beliefs (e.g., no one will like me why should I try to make friends), it becomes apparent that their social skills are not sufficient for engaging in desired social activities, or they have inconsistent motivation for initiating social contact. Techniques such as Cognitive Behavioral Therapy and Social Skills training might be used in tandem with environmental systems approaches, with the focus alternating between person and environmental systems targets in a dynamic fashion as environmental factors are successfully addressed and person-centered challenges wax and wane.

## Future Directions

A number of methods may be useful for studying the proposed bioecosystem network framework. Microsystems have been studied for decades using the well-validated sociogram method (57, 58). This approach entails guiding the individual through a structured interview, where they indicate which microsystem settings they actively participate in, which individuals are part of those settings, how often they interact with them, and whether those within their microsystem settings interact with each other. The sociogram can be administered and automatically scored via various online platforms, and may prove useful for testing the types of hypotheses presented below regarding the density and centrality of social microsystems. Exosystems are currently being explored via geocoding and digital phenotyping methods. For example, it is now possible to access government constructed databases (e.g., census) related to crime, poverty, neighborhood walkability, and transportation (59). These can be interfaced with digital maps and a region of interest can be set around an individual's address/location to evaluate whether exosystem factors are associated with symptoms (60). Similarly, macrosystem factors, such as ethnic density could also be studied using a combination of geolocation (where the participant is located) and publicly available statistics regarding the ethnic density of the location they are in at a given moment in time. Network analysis should be a valuable approach for analyzing these data streams and determining interactions within each system and how the various systems interact.

Investigators aiming to evaluate hypotheses regarding the onset of negative symptoms might consider study designs that are uncommon to the field of schizophrenia research and more prevalent in the field of developmental psychopathology. For example, to truly test developmental trajectory and hypotheses that negative symptoms can emerge in premorbid, prodromal, or chronic phases, one would need to use a prospective longitudinal design with a representative community sample starting in infancy or toddlerhood and followed them longitudinally throughout the most common later ages of onset seen in schizophrenia (e.g., mid to late 30's). Comprehensive assessments of person-level (e.g., genetics, neuroimaging) and environmental factors (e.g., via the sociogram, informant interviews, measures of built environment, home videos, digital phenotyping) would be needed at multiple timepoints to build latent class trajectory models. Studies aiming to examine whether environmental and person-level factors maintain negative symptoms once they already exist could utilize both cross-sectional or longitudinal designs using the aforementioned methods.

## Testable Hypotheses

Undoubtedly, some of the initial ideas posed here will be inaccurate and other elements that hold merit will need to be revised based on empirical evidence that emerges over time. This paper provides a starting point for systematically exploring negative symptoms from a bioecosystem perspective. Below are several initial testable hypotheses to guide these investigations, which propose that negative symptoms will be associated with:

1. Microsystem:
  - A reduced number of microsystem settings and reduced density of connections within settings.
  - Low ego centrality within microsystem settings (i.e., the individual will have less influence over activities and people within their microsystems).
  - Deprivation of resources within immediate environments that facilitate activity.
2. Mesosystem:
  - Reduced density of connections across microsystems.
  - High (others providing impetus for activities) or low (minimal interactions with others) centrality of key persons within the microsystem who enable or fail to facilitate interactions across microsystem settings, respectively.
3. Exosystem:
  - Deprivation of resources within indirect environments that facilitate activity (e.g., recreational centers, stores, transportation, local economy).
  - Greater influence of “power settings” that act on the individual and influence what transpires within microsystem settings and resources available (e.g., politics and laws influencing policies regarding disability benefits, subsidized housing, access to medical care).
4. Macrosystem:
  - Deficient knowledge of societal attitudes and norms about social, recreational, and goal-directed activities within their culture.
  - Incongruence between the culture of the individual and their microsystem settings (home, neighborhood) or current context (momentary location).
5. Developmental pathways:
  - Different components of the ecosystem throughout development/phase of illness.
  - Microsystems will carry greater weight at younger ages/earlier phases of illness when individuals are developing roles and skills.
  - Exosystem factors will exert greater influence as the individual becomes older and more independent.
  - The macrosystem will play an important role during early development, shaping an individual's knowledge of cultural norms for social, emotional, and motivated behaviors.
  - The mesosystem may be critical at all levels of development and phases of illness.
6. Transdiagnostic Generalizability:
  - Ecosystem factors in disorders outside the schizophrenia-spectrum; however, these influences will differ depending on the disorder and age/stage of development that negative symptoms emerged (e.g., autism vs. Parkinson's disease).

## Data Availability Statement

The original contributions presented in the study are included in the article/supplementary material, further inquiries can be directed to the corresponding author/s.

## Author Contributions

The author confirms being the sole contributor of this work and has approved it for publication.

## Conflict of Interest

The author declares that the research was conducted in the absence of any commercial or financial relationships that could be construed as a potential conflict of interest.
